# Text Messaging Interventions on Cancer Screening Rates: A Systematic Review

**DOI:** 10.2196/jmir.7893

**Published:** 2017-08-24

**Authors:** Catherine Uy, Jennifer Lopez, Chau Trinh-Shevrin, Simona C Kwon, Scott E Sherman, Peter S Liang

**Affiliations:** ^1^ Department of Medicine NYU School of Medicine New York, NY United States; ^2^ Department of Population Health NYU School of Medicine New York, NY United States; ^3^ Department of Medicine VA New York Harbor Manhattan Medical Center New York, NY United States

**Keywords:** text messaging, early detection of cancer, breast neoplasms, colorectal neoplasms, lung neoplasms, mHealth, uterine cervical neoplasms

## Abstract

**Background:**

Despite high-quality evidence demonstrating that screening reduces mortality from breast, cervical, colorectal, and lung cancers, a substantial portion of the population remains inadequately screened. There is a critical need to identify interventions that increase the uptake and adoption of evidence-based screening guidelines for preventable cancers at the community practice level. Text messaging (short message service, SMS) has been effective in promoting behavioral change in various clinical settings, but the overall impact and reach of text messaging interventions on cancer screening are unknown.

**Objective:**

The objective of this systematic review was to assess the effect of text messaging interventions on screening for breast, cervical, colorectal, and lung cancers.

**Methods:**

We searched multiple databases for studies published between the years 2000 and 2017, including PubMed, EMBASE, and the Cochrane Library, to identify controlled trials that measured the effect of text messaging on screening for breast, cervical, colorectal, or lung cancers. Study quality was evaluated using the Cochrane risk of bias tool.

**Results:**

Our search yielded 2238 citations, of which 31 underwent full review and 9 met inclusion criteria. Five studies examined screening for breast cancer, one for cervical cancer, and three for colorectal cancer. No studies were found for lung cancer screening. Absolute screening rates for individuals who received text message interventions were 0.6% to 15.0% higher than for controls. Unadjusted relative screening rates for text message recipients were 4% to 63% higher compared with controls.

**Conclusions:**

Text messaging interventions appear to moderately increase screening rates for breast and cervical cancer and may have a small effect on colorectal cancer screening. Benefit was observed in various countries, including resource-poor and non-English-speaking populations. Given the paucity of data, additional research is needed to better quantify the effectiveness of this promising intervention.

## Introduction

Cancer is a leading cause of death worldwide. The global burden of cancer is increasing in both developed and developing nations as the world population ages and established risk factors associated with economic development and urbanization become more prevalent, including smoking, obesity, sedentary lifestyle, and lower parity [1,2]. Worldwide, the most common malignancies are lung, colorectal, and prostate cancers for men and lung, colorectal, and breast cancers for women [3]. These cancers account for the vast majority of the cancer-related deaths and were responsible for more than 8 million deaths in 2012 [4].

In the United States alone, an estimated 1.7 million new cancer cases were diagnosed in 2016, resulting in nearly 600,000 deaths [5]. The cancer mortality rate in the United States has dropped by 23% since 1991. This trend is, in part, because of population-level screening tests, which detect early cancers and facilitate treatment before the disease becomes clinically apparent [6]. To date, screening tests for only four cancers have been shown to reduce mortality and the US Preventive Services Task Force (USPSTF) gives either an A or B grade recommendation for each. A grade A recommendation indicates a high certainty of substantial net benefit, and a grade B recommendation indicates a moderate to high certainty of at least moderate net benefit. Mammography reduces breast cancer mortality, and the USPSTF gives a B recommendation for biennial testing in average-risk women between 50 and 74 years of age [7]. Papanicolaou (Pap) testing and combined Pap and human papillomavirus (HPV) testing reduce the risk of death from cervical cancer in women aged between 21 and 65 years, which carries an A recommendation [8]. Colonoscopy, sigmoidoscopy, and fecal occult blood testing (FOBT) are the three tests that reduce colorectal cancer mortality, and the USPSTF gives an A recommendation to screen average-risk individuals in the age group of 50 to 75 years [9]. Finally, low-dose computed tomography (CT) has been shown to reduce lung cancer deaths in heavy smokers, and screening for these individuals aged 55 to 80 years carries a B recommendation [10]. Despite evidence-based guidelines for breast, cervical, colorectal, and lung cancers, there are challenges to increasing screening rates, particularly among segments of the population vulnerable to health disparities such as racial minorities and individuals from lower socioeconomic status. Tailored and targeted interventions to improve adoption and uptake of these guidelines are important to reducing overall mortality and disparities in outcome for these cancers.

As global economies and technology advance, more individuals have access to mobile phones. Text messaging, also known as short message service (SMS), is already being integrated into health care practices to improve adherence to contraception, smoking cessation, and weight loss programs, and it has been shown to increase attendance at primary care visits [11,12]. Several studies have demonstrated both patient interest in SMS reminders for cancer screening appointments and a positive impact on screening rates [13,14]. However, a cumulative analysis of the overall effect of text messaging on cancer screening rates has not been performed. The purpose of this systematic review was to assess the effect of text messaging interventions on increasing patient adherence to screening recommendations for breast, cervical, colorectal, and lung cancers.

## Methods

### Eligibility Criteria

We searched for clinical trials published from January 2000 to January 2017 that studied the effect of text messaging on screening for the four cancers of interest. We included all languages of publication. Types of screening methods were limited to mammography for breast cancer; Pap test and HPV test for cervical cancer; colonoscopy, FOBT, and sigmoidoscopy for colorectal cancer; and low-dose CT for lung cancer. Research subjects of all ages were considered. The outcome measures were absolute screening rates or relative screening rates in the text message group compared with a control group.

### Search Strategy

Studies were identified by searching several electronic databases and additionally searching references of relevant papers. Papers published in a language other than English were translated for review. The search was performed in PubMed, EMBASE, the Cochrane Library, Clinicaltrials.gov, Inspec, HSRProj (Health Services Research Projects in Progress), and NIH Reporter (Textbox 1). Attempts to identify gray literature were made by searching the BIOSIS Citation Index and Proquest Dissertations & Theses Global database, as well as by a manual search.

**Search terms for PubMed and comparable search terms for other databases.**

“Text Messaging”[MeSH] OR texting[tiab] OR “text messaging”[tiab] OR “text message”[tiab] OR “SMS message”[tiab] OR ((mobile OR cell OR cellular OR smart OR app OR application) AND phone[tiab]) AND (“Early Detection of Cancer”[MeSH] OR (cancer OR neoplasm OR neoplasms) AND screening) OR “Colonoscopy”[MeSH] OR “Colonoscopy”[tiab] OR “Colorectal Neoplasms/diagnosis”[MeSH] OR “Occult Blood”[MeSH] OR “fecal occult blood test”[tiab] OR “fecal occult blood testing”[tiab] OR “colon cancer screening” OR “colorectal cancer screening” OR “Mammography”[MeSH] OR mammography OR mammogram OR “Breast Neoplasms/diagnosis”[MeSH] OR “breast cancer screening” OR “Human Papillomavirus DNA Tests”[MeSH] OR “hpv test” OR “hpv testing” OR “Papanicolaou Test”[MeSH] OR “pap smear” OR “pap test” OR “Uterine Cervical Neoplasms/diagnosis”[MeSH] OR “Cervical Intraepithelial Neoplasia/diagnosis”[MeSH] OR “cervical cancer screening” OR “Lung Neoplasms/diagnosis”[MeSH] OR “Tomography, X-Ray Computed”[MeSH] OR “Tomography, Emission-Computed”[MeSH] OR “low dose CT” OR “low dose computed tomography” OR “low dose computed tomographic” OR “lung cancer screening”

### Selection and Quality Assessment of Studies

Citations were independently assessed by 2 investigators (CU and JL). Discrepancies were resolved by consensus and, when needed, with input from a third investigator (PSL). For studies requiring further clarification, the corresponding authors were contacted. The quality of the studies was evaluated using the Cochrane risk of bias tool. Extracted information included the following: (1) characteristics of the participants (including age and sex); (2) type of intervention (including test, duration of follow-up, and number of text messages sent); (3) outcome measure (including screening rates, odds ratio [OR], hazard ratio [HR], and confidence interval [CI] for unadjusted and adjusted results); (4) secondary endpoints; and (5) study limitations.

## Results

### Study Selection

**Figure 1:**
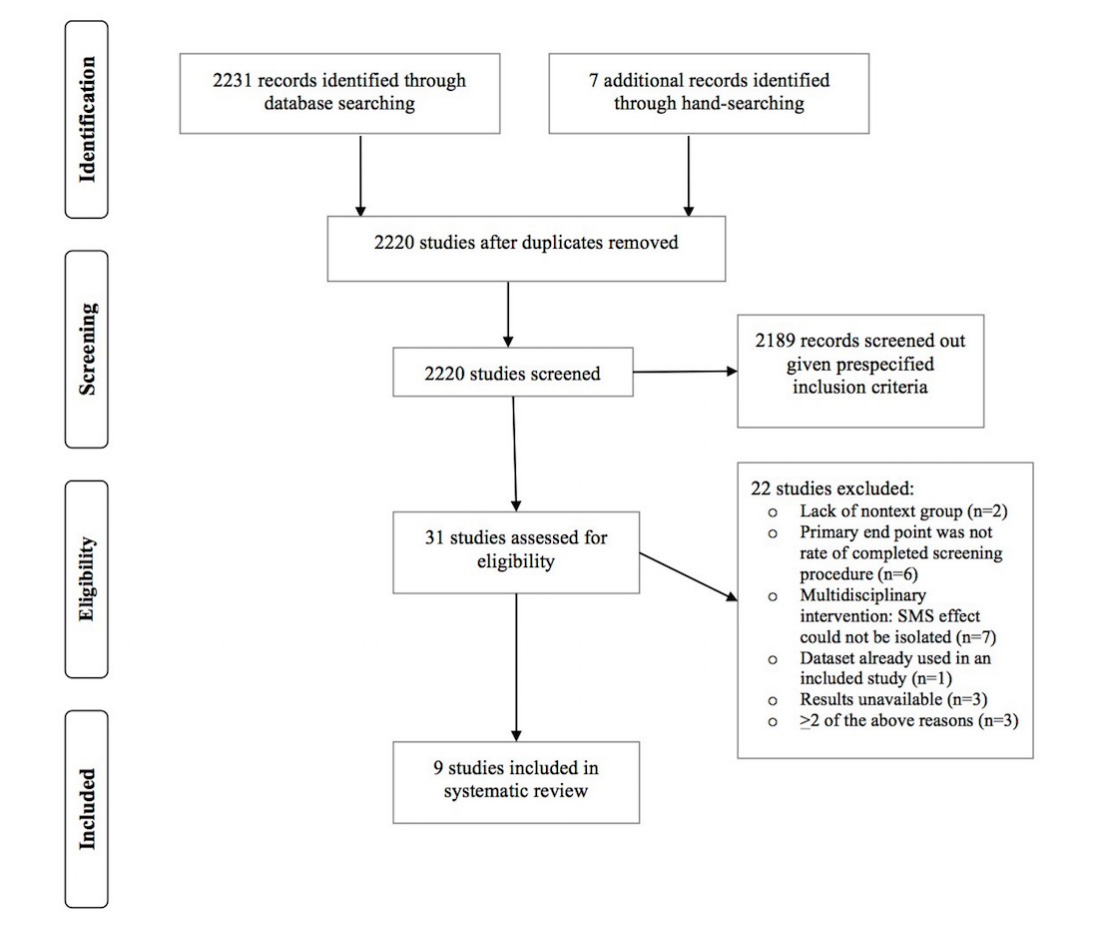
Flowchart of study selection process.

Our search yielded 2238 citations. After removing duplicates and screening abstracts, 31 papers underwent full review, 22 of which did not meet our prespecified criteria. A total of nine papers were included in the systematic review (Figure 1). These studies were based in England, Spain, Malaysia, Israel, and the United States. Five studies analyzed rates of breast cancer screening by mammography, one analyzed rates of cervical cancer screening by Pap test, and three studies measured rates of colorectal cancer screening (two by FOBT alone and one by a combination of colonoscopy, flexible sigmoidoscopy, or FOBT). No studies were found for lung cancer screening. Together, the included studies enrolled 77,099 participants, most of whom were women aged between 20 and 75 years (Tables 1 and 2). The length of follow-up from intervention delivery to outcome measurement ranged from 2 days to 6 months. Seven studies used a single or set of text messages delivered in 1 day [15-21]. The remaining trials delivered text messages over differing periods: one delivered several text messages over 7 days, and the other delivered individual texts at 1-month intervals until screening occurred (up to three texts maximum; [22,23]). Absolute screening rates were reported in all studies, and we compared the rates in intervention and control groups using the chi-square test (Table 3). Six studies also reported relative risk estimates using OR or HR (Table 4; [17-21,23]).

**Table 1:** Characteristics of studies included in the review of effect of text messaging interventions on cancer screening.

Author	Year	Cancer type	Screening test	Country	Study design	Publication type
Arcas [15]	2014	Breast	Mammogram	Spain	Randomized	Journal article
Icheku [16]	2015	Breast	Mammogram	England	Nonrandomized	Journal article
Kerrison [17]	2015	Breast	Mammogram	England	Randomized	Journal article
Lee [22]	2016	Breast	Mammogram	United States (Minnesota)	Randomized	Conference abstract
Vidal [18]	2014	Breast	Mammogram	Spain	Nonrandomized	Journal article
Abdul Rashid [19]	2013	Cervical	Pap test	Malaysia	Randomized	Journal article
Hagoel [20]	2016	Colorectal	FOBT	Israel	Randomized	Journal article
Hirst [21]	2016	Colorectal	FOBT	England	Randomized	Conference abstract
Muller [23]	2016	Colorectal	FOBT, flexible sigmoidoscopy, colonoscopy	United States (Alaska)	Randomized	Journal article

**Table 2:** Additional characteristics of studies included in the review of effect of text messaging interventions on cancer screening

Author	Screening test	N (text/control)	Age range	Intervention vs control groups	Follow-up
Arcas [15]	Mammogram	703 (470/233)	50-69	1 text message plus letter vs letter only	2 mo
Icheku [16]	Mammogram	2004 (552/1452)	50-70	1 text message plus letter vs letter only	1 wk
Kerrison [17]	Mammogram	2240 (1122/1118)	47-53	1 text message vs no reminder	2 d
Lee [22]	Mammogram	120 (60/60)	>40	Individualized text messages sent over 7 days vs informational brochure	6 mo
Vidal [18]	Mammogram	12,786 (3719/9067)	50-69	1 text message plus letter vs letter only	4.5 mo
Abdul Rashid [19]	Pap test	500 (250/250)	20-65	1 text message vs letter (two nonpertinent interventions were excluded from this review)	2 mo
Hagoel [20]	FOBT	48,091 (38,489/9602)	50-74	1 of 4 types of text message (I^a^, I+SC^b^, NI^c^, NI+SC) plus letter vs letter only	6 mo
Hirst [21]	FOBT	8269 (4134/4135)	60-74	1 text message if no screening occurred at 8 weeks vs usual care	4.5 mo
Muller [23]	FOBT, flexible sigmoidoscopy, colonoscopy	2386 (1193/1193)	40-75	Individual texts sent 1 month apart until screening occurred (3 texts maximum) vs usual care	6 mo

^a^I: interrogative.

^b^SC: social context.

^c^NI: noninterrogative.

**Table 3:** Absolute screening rates in text messaging versus control groups.

Author	Screening test	N (text/control)	Screening rate in text group, %	Screening rate in control group, %	Absolute increase in screening with text intervention, % (*P* value^a^)
Arcas [15]	Mammogram	703 (470/233)	81.3 (353/434)	76.8 (159/207)	+4.5 (*P*=.18)
Icheku [16]	Mammogram	2004 (552/1452)	68.1 (376/552)	60.47 (878/1452)	+7.6 (*P*=.002)
Kerrison [17]	Mammogram	2240 (1122/1118)	64.35 (722/1122)	59.12 (661/1118)	+5.3 (*P*=.01)
Lee [22]	Mammogram	120 (60/60)	40 (24/60)	25 (15/60)	+15.0 (*P*=.08)
Vidal [18]	Mammogram	12,786 (3719/9067)	74.91 (2786/3719)	65.00 (5894/9067)	+9.9 (*P*<.001)
Abdul Rashid [19]	Pap test	500 (250/250)	32.9 (54/164)	23.9 (47/197)	+9.1 (*P*=.05)
Hagoel [20]	FOBT	48,091 (38,489/9602)	9.78 (942/9631)	8.44 (817/9602)	+1.2 (*P*<.001)
Hirst [21]	FOBT	8269 (4134/4135)	40.49 (1674/4134)	39.85 (1648/4135)	+0.6 (*P*=.55)
Muller [23]	FOBT, flexible sigmoidoscopy, colonoscopy	2386 (1193/1193)	15.17 (181/1193)	11.90 (142/1193)	+3.3 (*P*=.02)

^a^Two-tailed *P* values based on chi-square test calculations using raw data from each study. Differences between reported (shown in text) and calculated (shown in table) *P* values are explained by differences in testing assumptions.

**Table 4:** Relative screening rates in text messaging versus control groups.

Author	Screening test	N (text/control)	Unadjusted OR^a^/HR^b^ (95% CI)	Adjusted OR/HR (95% CI)	Adjusted variables
Kerrison [17]	Mammogram	2240 (1122/1118)	1.26 (1.05-1.48)	1.25 (1.05-1.48)	Age, socioeconomic status
Vidal [18]	Mammogram	12,786 (3719/9067)	1.63 (1.49-1.78)	1.56 (1.43-1.70)	Age
Abdul Rashid [19]	Pap test	500 (250/250)	1.20 (0.76-1.87)	-	-
Hagoel [20]	FOBT	48,091 (38,489/9602)	-	I^c^: 1.17 (1.06-1.29) I+SC^d^: 1.24 (1.12-1.36) NI^e^: 1.09 (0.99-1.21) NI+SC: 1.14 (1.04-1.26)	Type of text message, age, gender, socioeconomic status
Hirst [21]	FOBT	8269 (4134/4135)	1.04 (0.95-1.13)	-	-
Muller [23]	FOBT, flexible sigmoidoscopy, colonoscopy	2386 (1193/1193)	1.30 (1.04-1.62)	-	-

^a^OR: odds ratio.

^b^HR: hazards ratio.

^c^I: interrogative.

^d^SC: social context

^e^NI: noninterrogative.

### Results and Quality of Individual Studies

#### Breast Cancer Studies

Arcas et al [15] assessed the impact of a single text message reminder on mammogram uptake in a single-blinded randomized control trial (RCT) over a 2-month period. The study found that women who received the SMS intervention were 4.5% more likely to undergo breast cancer screening (81.3%, 353/434, vs 76.8%, 159/207; *P*=.21). In subgroup analyses, those in the low secondary education level who received text messaging reported significantly higher screening, whereas results by age group were inconsistent. Women who were enrolled in the study were 3 times more likely to have participated in previous screening than women who were not enrolled (75.1%, 528/703, vs 24.55%, 488/1988), which may limit the generalizability of the findings. The high baseline screening rate may have also reduced the ability to detect a significant effect from the intervention.

Icheku and Arowobusoye [16] assessed how a single SMS sent 1 week before screening affected mammogram uptake. This non-RCT found that women who received text message reminders underwent mammograms 7.6% more than controls (68.1%, 376/552, vs 60.47%, 878/1452). The study determined the intervention and control groups based on whether individuals had a valid mobile number. Additionally, baseline socioeconomic characteristics of the two groups were not reported. The definition of screening was stringent and excluded any mammograms that occurred earlier or later than the scheduled appointment. Although the authors only reported descriptive statistics, we show the results of formal statistical testing in Table 3.

Kerrison et al [17] studied the effect of a single text message sent 2 days before a scheduled breast cancer screening appointment and measured the outcome using an electronic system. This single-blinded RCT found that women in the SMS group had a 5.3% absolute increase in mammogram uptake compared with controls (64.35%, 722/1122, vs 59.12%, 661/1118) in the intention-to-treat (ITT) analysis, corresponding to a 26% increase in the relative screening rate (OR 1.26, 95% CI 1.05-1.48). Of note, only about 40.64% (456/1122) of the participants in either group had a valid mobile phone number, and the per-protocol analysis of individuals with valid mobile numbers showed an 11.9% difference in screening uptake (71.7%, 327/456, vs 59.8%, 260/435). In multivariable logistic regression analysis, individuals residing in the most socioeconomically deprived areas were half as likely to attend screening as those from the least deprived areas (OR 0.53, 95% CI 0.35-0.80). This study was conducted at a single site and was limited to women who were screening-naïve, and therefore, it may not be generalizable to all women.

Lee et al [22] tested the impact of a 7-day course of individualized text messages on breast cancer screening rates. This single-blinded RCT found that after 6 months, there was a 15% higher absolute screening rate in participants who received the SMS intervention compared with those who received an informational brochure on breast cancer screening (40%, 24/60, vs 25%, 15/60). This difference had a reported *P*<.05 using a one-tailed test, whereas our two-tailed test showed *P*=.08 (Table 3). This trial had the smallest sample size of all studies included in this review. Additionally, text messages were individualized and culturally tailored for Korean-American women, which may limit the generalizability of the findings.

Vidal et al [18] assessed the effect of a single SMS on mammography rates after a follow-up of 4.5 months. Outcome was determined using a program database. Similar to the study by Icheku and Arowobusoye, this was also a non-RCT that allocated women to the intervention versus control group based on availability of mobile phone numbers. The authors found that text message recipients had a 9.9% higher absolute screening rate compared with controls (74.91%, 2786/3719, vs 65.00%, 5894/9067), corresponding to a 63% higher relative screening in the intervention group (OR 1.63, 95% CI 1.49-1.78). The odds were 56% higher after adjusting for age (OR 1.56, 95% CI 1.43-1.70). In a stratified analysis based on previous screening behavior and geography, the effect of SMS reminders was greatest among women who had not undergone previous screening and were living in sparsely populated areas where postal service is unreliable. A limitation of the nonrandomized study design is that women with and without registered mobile numbers may have baseline differences in health care access and health literacy.

#### Cervical Cancer Studies

Abdul Rashid et al [19] assessed the effect of a text message intervention on Pap testing after a 2-month follow-up in a single-blinded RCT. In the per-protocol analysis, those who received SMS had a 9.1% increase in absolute screening rate compared with those who received a letter containing the same information (32.9%, 54/164, vs 23.9%, 47/197; *P*=.05). In the ITT analysis, there was a 20% higher relative screening rate (OR 1.20, 95% CI 0.76-1.87). The study only included women who had a previously normal Pap and therefore may not be generalizable to women who are screening-naïve.

#### Colorectal Cancer Studies

Hagoel et al [20] assessed how the language and content of SMS reminders affected FOBT completion. Participants were randomized to one of five groups. The four intervention groups received one of four types of text reminders: messages were either framed as a question (interrogative) or a statement (noninterrogative) and additionally either included or excluded a reference to peer screening behavior (social context). Controls did not receive a text intervention. Outcomes were assessed after 6 months using a national database. The authors found that 9.78% (942/9631) of patients who received any text intervention completed FOBT, compared with 8.44% (817/9602) of those who did not receive a text message. This corresponded to a 9% to 24% higher odds of screening in the intervention groups, which were statistically significant for all groups except the one that received noninterrogative messages without social context. In multivariable logistic regression analysis, participants who were older than 60 years (OR 1.13, 95% CI 1.06-1.20), female (OR 1.21, 95% CI 1.13-1.28), and of low socioeconomic status (OR 1.19, 95% CI 1.10-1.30) were more likely to undergo screening. This study excluded patients who were screening-naïve, which limits its generalizability. In addition, participants received an invitation to order or pick up a FOBT kit rather than the kit itself, which may present an additional barrier to screening.

Hirst et al [21] also assessed colorectal cancer screening using mailed FOBT kits. The intervention group received a single SMS reminder if the FOBT kit was not returned after 8 weeks from the initial invitation. The ITT analysis at 18 weeks showed that text messaging reminders did not significantly increase the number of individuals screened, with FOBT kit return rates of 40.49% (1674/4134) in the intervention group and 39.85% (1648/4135) in the control group (OR 1.04, 95% CI 0.95-1.13). However, mobile phone numbers were available for only 49.44% (4089/8269) of the study participants in either group, even though all participants were included in the analysis. Of those who actually received the SMS reminder, 18.42% (189/1026) participated in screening. Additionally, a subgroup analysis of the first-time screening group found a 5.6% higher absolute screening rate in the intervention group compared with controls (34.9%, 282/809, vs 40.5%, 297/733; OR 1.31, 95% CI 1.00-1.71). No difference in uptake was observed by sex, index of deprivation, or age.

Finally, Muller et al [23] examined text messaging and colorectal cancer screening in Alaskan Natives. This single-blinded RCT sent individuals up to three text messages at 1-month intervals and assessed the outcome from electronic health records after 6 months of follow-up. The study found a 3.3% absolute increase in colorectal cancer screening, using colonoscopy, sigmoidoscopy, or FOBT. This corresponded to a 30% increase in the relative screening rate (HR 1.30; 95% CI 1.04-1.62). Notably, higher screening rates were only observed in women who received the intervention, and no statistically significant change was observed in men. The single site and specific population of this study may limit its generalizability.

A common limitation of the included studies was a relatively short duration of follow-up, which may have resulted in an underestimation of the text messaging effect, especially if there were substantial delays in the scheduling of screening tests. Overall, potential biases in study design and the focus on specific and often homogeneous populations for many of the studies may limit the generalizability of the results. Two studies were published as abstracts and may not have undergone a similar peer review process as the full manuscripts [21,22]. Two other studies were nonrandomized trials that assigned individuals to intervention or control groups based on the availability of a mobile phone number, which raises the concern that the groups may be different in other characteristics relevant to screening [16,18]. None of the studies formally assessed health literacy. Finally, with the exception of the Hagoel et al study [20], none of the included trials used a theoretical framework in the text messaging interventions.

## Discussion

### Principal Findings

In this systematic review of text messaging interventions and evidence-based cancer screening, we found that SMS can moderately increase screening rates for breast and cervical cancers and may improve colorectal cancer screening to a lesser degree. Across all studies, text messaging interventions led to increases in absolute screening rates of 0.6% to 15% and relative screening rates of 4% to 63%. Given the small number of studies and heterogeneity in design, we did not attempt to quantify the overall effect of SMS on cancer screening using meta-analysis.

Of the six studies that examined text messaging in breast and cervical cancer screening, increases in absolute screening rates ranged from 4.5% to 15%, and the three studies that reported relative screening rates found improvements of 20% to 63%. Although the smallest reported change in absolute screening rate was not statistically significant (Arcas et al, 4.5%), both the overall direction and the magnitude of absolute effect for SMS seem consistent for breast and cervical cancers. However, as only one cervical cancer screening study met our inclusion criteria, confirmatory investigations are clearly needed.

For colorectal cancer screening, the three included studies found much smaller effects on absolute screening rates, with increases ranging from 0.6% to 3.3%. Hirst et al [21] found a 0.6% difference in absolute screening and a nonsignificant 4% increase in relative screening with FOBT, but this almost certainly underestimates the true impact of SMS because only 24.81% (1026/4134) of the intervention group actually received a text message reminder. Hagoel et al [20] conducted the largest study included in this review, and the results suggest that how the message is phrased has important implications for how it is received: messages posed as a question were more effective than those phrased as a statement, and the most effective messages were questions accompanied by social context. Although the 1.2% increase in absolute screening rate is small, it should be noted that participants in the study had to first order and then complete the FOBT kit, and it has been shown that directly mailing kits to patients significantly improves screening rates [24]. The study of Alaskan Natives by Muller et al [23] was the only colorectal cancer screening study to include colonoscopy and flexible sigmoidoscopy as well as FOBT, and the 3.3% increase in absolute screening rate is more impressive after factoring in that every member of the health care system already receives telephone and letter reminders as well as in-office physician referrals for screening. Whether SMS interventions are more or less effective for colonoscopy than FOBT is an important question, because colonoscopy has become the predominant screening test in the United States and remains the requisite follow-up test for all abnormal FOBTs.

There are at least two reasons why the effectiveness of SMS may differ for colorectal cancer screening and breast or cervical cancer screening. The first may be a difference in the complexity or acceptability of the screening test. Although mammography and the Pap test simply require patients to attend a clinic appointment, patients must be willing to collect one or more stool specimens to complete a FOBT. Colonoscopy is even more complicated, as it requires patients to restrict their diet for several days, drink a large volume of fluids for bowel preparation, and finally undergo an invasive examination. A few trials have studied the impact of text messaging on the quality of bowel preparation, but none has measured the effect of bowel preparation instructions via SMS on screening rates themselves [25,26]. If the problem lies with a higher barrier to overcome for colorectal versus breast and cervical cancer screening, then the strategies used in some included studies may help to maximize the effect of SMS. Providing culturally tailored messages, as both Lee et al and Muller et al did, may be more impactful than generic messages. Similarly, Hagoel et al demonstrated the additional value of using interrogative and contextualized messages. The language of text messaging will continue to be an important area of research, especially for complex screening procedures such as colonoscopy that may require instructions as well as reminders.

A second reason may be the gender difference in the study populations, as the cervical and breast cancer study participants were all women. Indeed, perhaps the most striking finding from the Muller et al study was that only women benefited from the intervention. Hagoel et al also found that women who received the text messages had a statistically significant 21% greater odds of FOBT uptake than men (OR 1.21; 95% CI 1.13-1.28 [20]). However, Hirst et al found no significant difference in FOBT uptake between men and women. It is unclear whether true gender differences exist in how text messages are received or how it motivates behavioral change. Additional studies on the influence of SMS on colorectal cancer screening will help to better quantify its effect for each of the various screening tests, as well as any differences that may exist with respect to gender.

There are many clear advantages to using text messaging to increase cancer screening. According to the United Nations, in 2013, nearly 6 billion of the world’s 7 billion people possessed mobile phones, compared with the 4.5 billion who had access to toilets or latrines [27]. These statistics have clear implications for the potential of using mobile technology to reach underserved communities, including those living in rural and underdeveloped areas [28,29]. A 2016 survey by the National Center for Health Statistics found that 49% (18,074/36,885) of all US adults live in households with only wireless telephones, and those living in (63%, 23,274/36,885) or near (54%, 19,918/36,885) poverty were more likely to live in wireless-only households than those with higher income (48.2%, 17,779/36,885) [30]. In addition, all minority racial groups were more likely to live in wireless-only households, with the greatest difference being between whites and those of Hispanic or Latino descent (45.0%, 16,598/36,885 vs 63.7%, 23,496/36,885). For populations with low health literacy, the short and simple format of text messages may also improve comprehension and reduce a formidable barrier to access. Although the studies included in this review did not specifically report results in participants with low health literacy, text messaging has been shown to improve medication adherence in this population [31].

Beyond the prevalence of mobile phone usage and its reach in low-income settings, another benefit of utilizing text messages for patient outreach is the ability to directly send discreet information in real time, which can then be accessed at the patient’s convenience. This delivery method does not require patients to be available at a particular time or place to receive information, as a telephone call would. Texting also does not rely on an updated or even stable address, as a letter would. However, SMS does require a stable phone number and also may raise concerns about confidentiality if multiple family members share a mobile phone. The ability of SMS to connect with remote and socioeconomically disadvantaged populations is supported by several of the included studies. The mammography study by Vidal et al [18] showed that women who lived in areas with less reliable postal service benefited more from the SMS intervention. Hagoel et al found higher rates of FOBT uptake in patients of low versus high socioeconomic status (OR 1.19; 95% CI 1.10-1.30 [20]). Finally, Kerrison et al [17] found that text messaging was particularly effective at increasing screening rates for patients from the lowest socioeconomic category, who saw a 13.6% absolute increase in screening from the control to intervention group. Vidal et al also demonstrated that adding text messages to an invitation letter was cost-effective for breast cancer screening, and similar conclusions have been reached about the cost-effectiveness of text messaging to improve other health care outcomes [32,33].

Text messaging has been found to be effective for promoting a variety of beneficial health behaviors. Whittaker et al [34] conducted a meta-analysis of text messaging interventions for smoking cessation and found a 67% higher likelihood of successful cessation at 6 months compared with controls. Arambepola et al [35] analyzed 13 trials of diabetic patients and found a statistically significant 0.53% decrease in hemoglobin A1c as well as a nonsignificant 0.25 kg/m^2^ reduction in body mass index in patients who received an SMS intervention. Two meta-analyses have shown that text messaging also improves attendance at primary care and hospital outpatient clinic appointments by 14% to 48% compared with controls [36,37]. Together, these studies consistently convey text messaging’s efficacy as an intervention for health behavioral change, which is further supported by our analysis of cancer screening.

Some studies did not meet our inclusion and exclusion criteria but merit mentioning. Oakley-Girvan et al [38] found that text messaging decreases time to follow-up after abnormal mammograms in a Spanish-speaking Latina population, suggesting that text messaging may be useful to increase rates of both regular screening and follow-up for management of abnormal results. This study also suggests the utility of text messaging for communicating in patients’ preferred language, potentially overcoming communication barriers between physicians and patients. For colorectal cancer, a multifaceted intervention that included text messaging in addition to a mailed letter, instructions on using the stool test, paid return envelopes, and phone messages increased rates of FOBT screening for colorectal cancer from 37% (84/225) to 82% (185/225) [39]. However, we excluded this study because the effect of text messaging could not be isolated.

**Table 5:** Ongoing studies evaluating the impact of text messaging interventions on cancer screening rates.

Author	Cancer type	Country	Study title
Huf S	Cervical cancer	England	Can Text Reminders Improve Uptake of Cervical Screening?
Palafox N	Cervical cancer	United States (Hawaii)	Pilot Project 1: Reducing Cervical Cancer Screening Health Disparities Among Pacific Islanders Living in Guam (GU) and Hawaii (HI)
Baker D	Colorectal cancer	United States (Illinois)	Improving Rates of Repeat Colorectal Cancer Screening
Baker D	Colorectal cancer	United States (Illinois)	Improving Rates of Colorectal Cancer Screening Among Never Screened Patients
Ma G	Colorectal cancer	United States (Pennsylvania)	A Multilevel CBPR^a^ Intervention to Improve Colorectal Cancer Screening in Underserved Vietnamese Americans
Smith J	Colorectal cancer	United States (Michigan, New Mexico, Washington)	Evaluation of an Intervention to Increase Colorectal Cancer Screening in Primary Care Clinics

^a^CPBR: Community-based participatory research.

Notably, our search did not find any studies that assessed the effect of text messaging on screening for lung cancer, a leading cause of cancer death in the United States that affects both men and women. This is not entirely surprising, given the relatively new guidelines that support screening for the subset of the population with a substantial smoking history. However, it highlights a knowledge gap in the relationship between an effective behavioral intervention and a screening test that has been proven to save lives in heavy smokers, and further studies on the subject are warranted.

Several trials are currently under way that may further quantify the relationship between text messaging and cancer screening (Table 5). In addition, as seven of the nine studies we found were conducted in Europe and Asia, more domestic research is needed to ensure that the results are applicable to the US population.

### Limitations

Although we developed a comprehensive search strategy to query the relevant literature, it is possible that some studies were not identified. There was a paucity of data that met our inclusion criteria, and we found only one study on cervical cancer and three on colorectal cancer. In addition, there was substantial heterogeneity in study design, content of the text message intervention, as well as time to follow-up in the included studies. For these reasons, we did not perform a meta-analysis.

### Conclusions

A systematic review of the literature suggests that text messaging interventions can increase screening rates for breast and cervical cancers to a moderate degree and for colorectal cancer to a smaller degree. Implementation of text messaging for cancer screening may be an effective method to increase screening rates and thereby decrease cancer-related mortality, even in resource-poor and non-English-speaking populations. However, additional research is needed to determine whether these results apply to all cancer screening tests at the population level.

## Abbreviations

CT: computed tomography
FOBT: fecal occult blood test
HPV: human papillomavirus
HR: hazard ratio
ITT: intention-to-treat
I: interrogative
NI: noninterrogative
OR: odds ratio
RCT: randomized controlled trial
SC: social context
SMS: short message service
USPSTF: US Preventive Services Task Force
